# Methods for evaluating clustering algorithms for gene expression data using a reference set of functional classes

**DOI:** 10.1186/1471-2105-7-397

**Published:** 2006-08-31

**Authors:** Susmita Datta, Somnath Datta

**Affiliations:** 1 Department of Bioinformatics and Biostatistics, University of Louisville, Louisville, KY 40202, USA

## Abstract

**Background:**

A cluster analysis is the most commonly performed procedure (often regarded as a first step) on a set of gene expression profiles. In most cases, a post hoc analysis is done to see if the genes in the same clusters can be functionally correlated. While past successes of such analyses have often been reported in a number of microarray studies (most of which used the standard hierarchical clustering, UPGMA, with one minus the Pearson's correlation coefficient as a measure of dissimilarity), often times such groupings could be misleading. More importantly, a systematic evaluation of the entire set of clusters produced by such unsupervised procedures is necessary since they also contain genes that are seemingly unrelated or may have more than one common function. Here we quantify the performance of a given unsupervised clustering algorithm applied to a given microarray study in terms of its ability to produce biologically meaningful clusters using a reference set of functional classes. Such a reference set may come from prior biological knowledge specific to a microarray study or may be formed using the growing databases of gene ontologies (GO) for the annotated genes of the relevant species.

**Results:**

In this paper, we introduce two performance measures for evaluating the results of a clustering algorithm in its ability to produce biologically meaningful clusters. The first measure is a biological homogeneity index (BHI). As the name suggests, it is a measure of how biologically homogeneous the clusters are. This can be used to quantify the performance of a given clustering algorithm such as UPGMA in grouping genes for a particular data set and also for comparing the performance of a number of competing clustering algorithms applied to the same data set. The second performance measure is called a biological stability index (BSI). For a given clustering algorithm and an expression data set, it measures the consistency of the clustering algorithm's ability to produce biologically meaningful clusters when applied repeatedly to similar data sets. A good clustering algorithm should have high BHI and moderate to high BSI. We evaluated the performance of ten well known clustering algorithms on two gene expression data sets and identified the optimal algorithm in each case. The first data set deals with SAGE profiles of differentially expressed tags between normal and ductal carcinoma in situ samples of breast cancer patients. The second data set contains the expression profiles over time of positively expressed genes (ORF's) during sporulation of budding yeast. Two separate choices of the functional classes were used for this data set and the results were compared for consistency.

**Conclusion:**

Functional information of annotated genes available from various GO databases mined using ontology tools can be used to systematically judge the results of an unsupervised clustering algorithm as applied to a gene expression data set in clustering genes. This information could be used to select the right algorithm from a class of clustering algorithms for the given data set.

## Background

The primary purpose of this paper is to introduce two new external indices for measuring the performance of a clustering algorithm for the specific purpose of grouping genes using their expression profiles.

Clustering of genes on the basis of expression profiles is a frequently, if not always, performed operation in analyzing the results of a microarray or SAGE study. Often times it is taken as a first step in understanding how a class of genes act in consort during a biological process. Statistics and machine learning literature provide a huge choice of clustering tools for such unsupervised learning operations. Not only do multiple algorithms exist, but even a single algorithm may rely on various user selectable tuning parameters such as desired number of clusters, or threshold values for forming a new cluster, initial values etc. Naturally, the results may be quite varied (see, e.g., [1-3]). Although, the hierarchical clustering method UPGMA [4] is used most often with microarray data sets (partly due to its early integration into existing software), the following algorithms are also generally considered to be solid performers in the clustering world and are freely available through various R [5] libraries: a partition method called K-means [6], a divisive clustering method Diana [7], a fuzzy logic based method Fanny [7], neural network based methods SOM (self-organizing maps, [8]) and SOTA (self-organising tree algorithm, [9]) and a normal mixture model based clustering [10].

Past evaluations of clustering algorithms have been of general (non-biological) nature. For example, a good clustering algorithm ideally should produce groups with distinct non-overlapping boundaries, although a perfect separation can not typically be achieved in practice. Figure of merit measures (FOM, hereafter) [11] such as the silhouette width [12] or the homogeneity index [13] can be used to evaluate the (visual) separation of groups obtained from a clustering algorithm. The concept of stability of a clustering algorithm was taken into consideration in [3] (also see [14]). A resampling based validity scheme was proposed in [15].

Although popular statistical clustering algorithms (e.g., UPGMA) have often been reported to successfully produce clusters of functionally similar genes, it is important to make that requirement a part of the evaluation strategy in selecting one from a list of competing clustering algorithms. Some attempts in this direction have been made in recent years (e.g., [16-18]). These papers propose scoring a clustering algorithm based on the biological similarity of the resulting clusters in some fashion, although all of them ignore the stability issue. The index proposed in [16] is based on the idea of mutual information content between statistical clusters and biological attributes. The entropy is taken as a measure of information content and a filtered collection of all GO terms is used as attributes. [17] used an ANOVA based test of equality of means amongst the cluster members to define their validation index. One potential difficulty with this approach is that a quantitative conversion of biological attributes is needed (which may not be natural and may not preserve the information content). [18] used an information content technique proposed by [19] to compute their validation index. There also exists another set of papers (e.g., [20-22]) where the main objective is that of biological interpretation of the clusters produced by a clustering algorithm.

In this paper, we introduce two performance measures for evaluating the results of a clustering algorithm in its ability to produce biologically meaningful clusters. The first measure is a biological homogeneity index (BHI). As the name suggests, it is a measure of how biologically homogeneous the clusters are. This can be used to quantify the performance of a given clustering algorithm such as UPGMA in grouping genes for a particular data set and also for comparing the performances of a number of competing clustering algorithms applied to the same data set. The second performance measure is called a biological stability index (BSI). For a given clustering algorithm and an expression data set, it measures the consistency of the clustering algorithm's ability to produce biologically meaningful clusters when applied repeatedly to similar data sets. A good clustering algorithm should have high BHI and moderate to high BSI. We also provide an R-code with some simple illustrations for computing these indices [see Additional file 1]. We evaluated the performance of ten well known clustering algorithms using this dual measures approach on two gene expression data sets and identified the optimal algorithm in each case.

We use publicly available GO [23] tools and databases to obtain the functional information in our illustrative real data examples. They are used to produce a reference collection of functional classes with respect to which a clustering algorithm was judged for homogeneity and stability. In particular, it has no relations to the idea of co-clustering which uses statistical clustering within each GO term.

## Results

**Figure 1:**
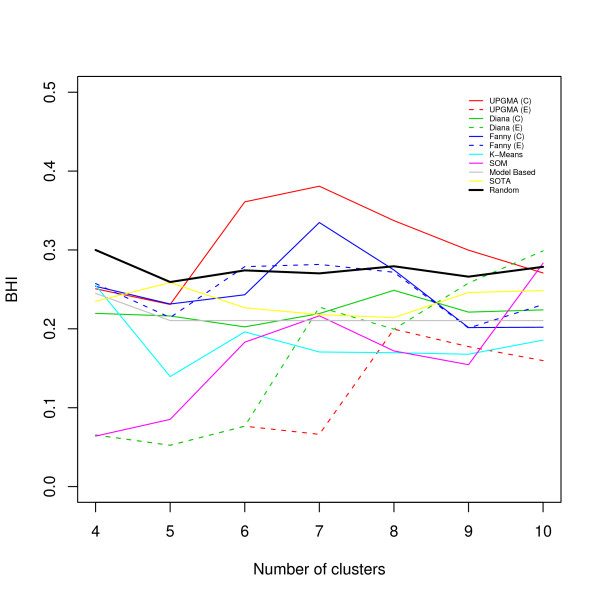
BHI for various clustering algorithms applied to the normal and DCIS samples in breast cancer data. The thick black line is the 95th percentile of BHI values under random clustering.

We first consider the breast cancer data. This data set consisted of expression profiles of 258 significant genes based on their eleven dimensional expression profiles over four normal and seven DCIS samples. Based on the size of the data set we judge that a cluster size between four and ten might be appropriate. Thus, both the biological homogeneity index (BHI) and the biological stability index (BSI) was computed for each clustering algorithm in this range of cluster numbers. As described in the Methods section, we used eleven functional classes for this study. Figure 1 shows the plots of BHI for the ten clustering strategies along with the results for random clustering. The thick black piecewise linear curve denotes the 95-th percentiles of the BHI values obtained by random clustering – these are computed by a Monte carlo scheme as described in the methods section based on 500 iterates. Thus, the probability of obtaining a value of BHI as high as that just by chance is estimated to be less than 5%. Therefore, any score higher than the thick black curve by a clustering algorithm will be judged to be "statistically significant".

Three of the seven clustering algorithms were used with two choices of dissimilarity measures. These are indicated by the line types with solid lines corresponding to one-minus the Pearson's correlation coefficient as a dissimilarity measure and dashed lines corresponding to Euclidean distance, respectively. In the rest of the paper, the term correlation refers to the Pearson's correlation coefficient. The plot of BHI reveals that UPGMA with the correlation measure happens to produce most homogeneous biological clusters based on this data set and the results are statistically significant when the number of clusters are between six and ten. We also computed p-values under a non-uniform resampling which maintains the same cluster sizes (on the average) as produced by a given clustering algorithm. This is easily accomplished by drawing a random sample with probability proportional to the original cluster sizes instead of a simple random sample in Step 2 of the statistical scoring algorithm. Note that it is computationally expensive however, since separate resampling needs to be done for each *k *and clustering algorithm combination. UPGMA with correlation and *k *between six and nine remains significant at 5% based on the non-uniform resampling as well (p-values were .028, .018, .030 and .046, respectively). Interestingly, the performance of most other clustering algorithms was not significantly better than random clustering except for Fanny with cluster size *k *= 7 (Fanny, Euclidean with *k *= 8 is borderline significant) and Diana (Euclidian) with *k *= 10.

**Figure 2:**
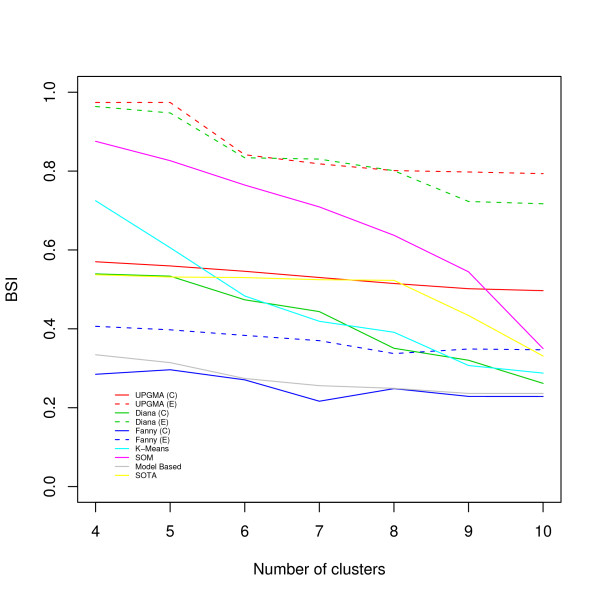
BSI for various clustering algorithms applied to the normal and DCIS samples in breast cancer data.

The biological stability index (BSI) is plotted in Figure 2. The 95th percentile BSI values under random clustering were all nearly zero and are not plotted further. We can say that all the clustering algorithms have produced significantly more consistent answers as compared to random clustering which is perhaps not too surprising. The fuzzy logic based clustering Fanny seems to be the least stable and UPGMA (Euclidian), along with Diana (Euclidian), seems to be the most stable in their capabilities of producing clusters using reduced data sets that are biologically alike. Considering both indices, we would say that UPGMA (correlation), which also has decent stability, is the best choice for this data set provided investigators select six to nine clusters where seven seems to be the optimal number of clusters to maximize the biological homogeneity. Diana (Euclidian) will be a worthwhile consideration if ten clusters are desired.

**Figure 3:**
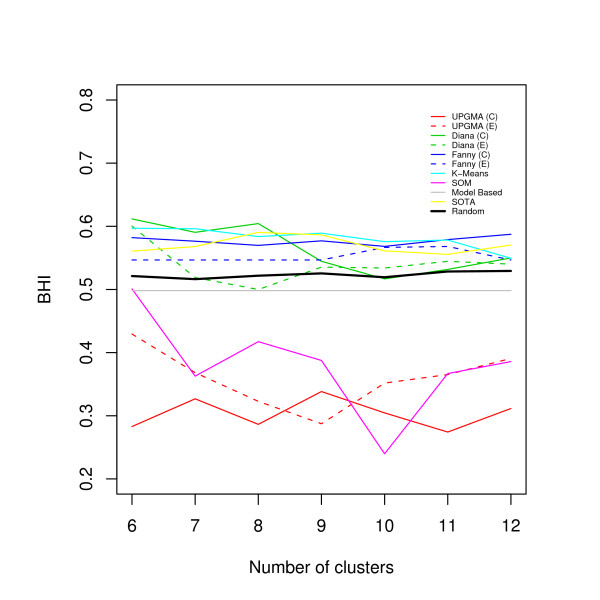
BHI for various clustering algorithms applied to the positively expressed genes in yeast sporulation data with functional classes from FatiGO. The thick black line is the 95th percentile of BHI values under random clustering.

**Figure 4:**
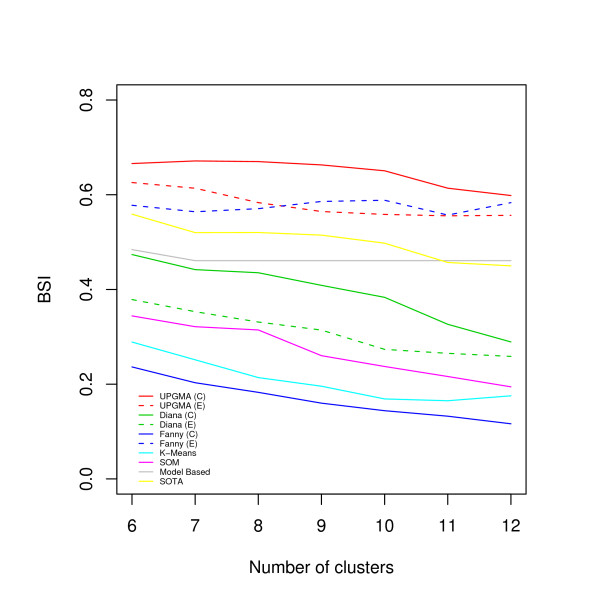
BSI for various clustering algorithms applied to the positively expressed genes in yeast sporulation data with functional classes from FatiGO.

**Figure 5:**
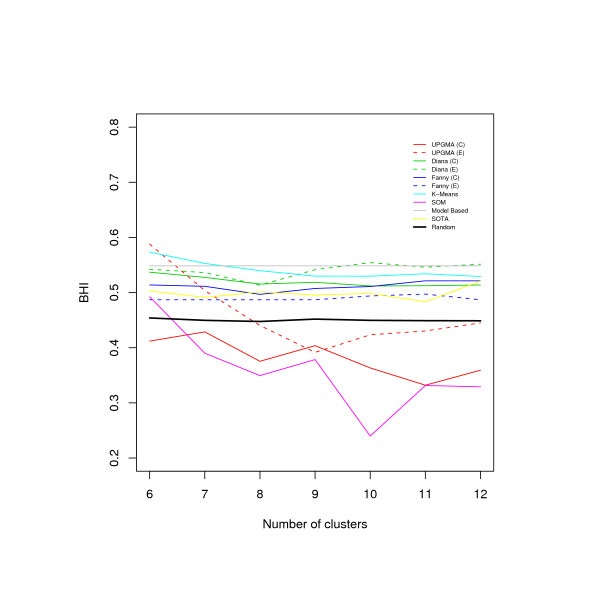
BHI for various clustering algorithms applied to the positively expressed genes in yeast sporulation data with functional classes from FunCat. The thick black line is the 95th percentile of BHI values under random clustering.

**Figure 6:**
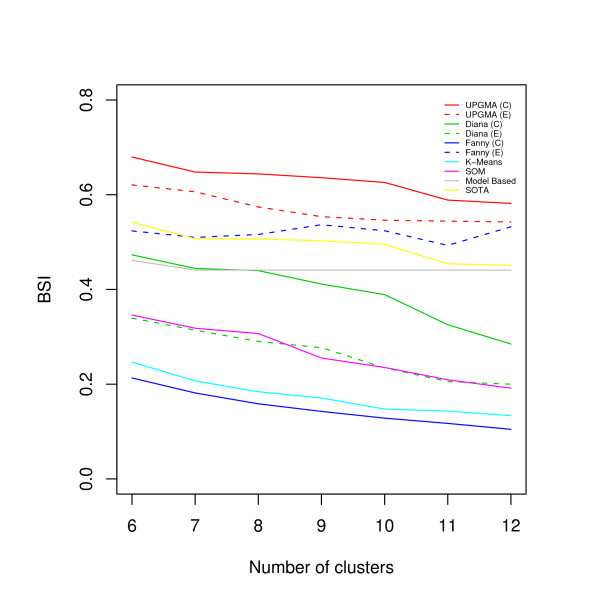
BSI for various clustering algorithms applied to the positively expressed genes in yeast sporulation data with functional classes from FunCat.

Next we report the results for the sporulation data set. As stated in the methods section, we have used two different sets of functional classes for biological validations. For the details, we refer to Figures 3 and 5 which show the biological homogeneity index (BHI) and Figures 4 and 6 which show the biological stability index (BSI) under the two functional schemes. A range of six to twelve was selected for the number of clusters. The plots of BHI show that for this data set, under both sets of functional classes, Fanny, Diana (correlation), K-Means and SOTA are doing well whereas UPGMA and SOM are not. Model based and Diana (Euclidean) perform well under the FunCat classes but not with respect to the FatiGO classes.

Model based selected only six clusters even if a larger maximum number of clusters was specified. The biological stability index, on the other hand was high for UPGMA and Fanny (Euclidian) but low for K-Means and Fanny (correlation). Thus, considering everything, Fanny (Euclidian) seems to be the optimal algorithm for the yeast data set. Other overall good performers were Diana (correlation) and SOTA.

## Discussions and conclusion

Historically, validation measures for clustering algorithms are based on the data themselves. They measure the extent of a clustering algorithms's ability in finding similarity structures hidden in the data. However, for clustering biological data such as the gene expression profiles, it would be reasonable to consider external measures that employ the existing biological knowledge (which can be taken as the "ground truth"). As argued by [24], internal measures by themselves may not be suitable for biological data which are often subject to many sources of noise (including experimental artifacts).

The two indices introduced here are useful in quantifying the results of an unsupervised clustering in grouping genes with similar biological functions given a reference collection of relevant functional classes. These indices will be preferable over internal indices when there is a substantive existing biological knowledge about the genome under consideration (e.g., as reflected by the proportion of annotated genes).

As mentioned in the background section, the stability aspect was absent in existing external indices based on biological information. In our earlier work [3], Diana (Euclidian) was recommended based on our internal stability measures and an external FOM measure called "distance from model profiles". It should be noted, however, that Diana (correlation) was not included for benchmarking in [3]. Based on the new external FOM, the biological homogeneity index BHI, both Diana (correlation) and (Euclidean) look good; however based on the new external stability measure BSI, Diana (correlation) is preferable over Diana (Euclidian).

Past studies have often concluded that clustering of the gene expression profiles (typically via UPGMA with correlation similarity) show that functionally similar genes are grouped together. This is often concluded by inspecting a handful of handpicked genes. Such conclusions are inherently incomplete unless one can quantify the agreement between the clusters produced via the expression profiles and the biological classes because it is likely that many biologically unrelated genes will be grouped together as well.

The proposed indices are easy to interpret and easy to implement. They are also useful in identifying the optimal clustering algorithm for a given data set in its ability to cluster biologically similar genes. As illustrated in this paper, no single clustering algorithm is likely to be the winner in all data sets. The approach introduced here will be even more useful as the gene ontology databases grow with time.

As shown with the illustrated data sets, the biological indices can also guide us to determine the number of clusters to be used in a clustering routine. Once an optimal algorithm is determined one may choose *k *that maximizes BHI for that algorithm in the given range. This approach would indicate that seven and eleven, respectively, are the optimal number of clusters to be used for the breast cancer data and the sporulation data.

## Methods

Suppose G
 MathType@MTEF@5@5@+=feaafiart1ev1aaatCvAUfKttLearuWrP9MDH5MBPbIqV92AaeXatLxBI9gBamrtHrhAL1wy0L2yHvtyaeHbnfgDOvwBHrxAJfwnaebbnrfifHhDYfgasaacH8akY=wiFfYdH8Gipec8Eeeu0xXdbba9frFj0=OqFfea0dXdd9vqai=hGuQ8kuc9pgc9s8qqaq=dirpe0xb9q8qiLsFr0=vr0=vr0dc8meaabaqaciaacaGaaeqabaWaaeGaeaaakeaaimaacqWFge=raaa@382D@ is the set of all genes for a given microarray experiment. Let C
 MathType@MTEF@5@5@+=feaafiart1ev1aaatCvAUfKttLearuWrP9MDH5MBPbIqV92AaeXatLxBI9gBamrtHrhAL1wy0L2yHvtyaeHbnfgDOvwBHrxAJfwnaebbnrfifHhDYfgasaacH8akY=wiFfYdH8Gipec8Eeeu0xXdbba9frFj0=OqFfea0dXdd9vqai=hGuQ8kuc9pgc9s8qqaq=dirpe0xb9q8qiLsFr0=vr0=vr0dc8meaabaqaciaacaGaaeqabaWaaeGaeaaakeaaimaacqWFce=qaaa@3825@_1_,.....,C
 MathType@MTEF@5@5@+=feaafiart1ev1aaatCvAUfKttLearuWrP9MDH5MBPbIqV92AaeXatLxBI9gBamrtHrhAL1wy0L2yHvtyaeHbnfgDOvwBHrxAJfwnaebbnrfifHhDYfgasaacH8akY=wiFfYdH8Gipec8Eeeu0xXdbba9frFj0=OqFfea0dXdd9vqai=hGuQ8kuc9pgc9s8qqaq=dirpe0xb9q8qiLsFr0=vr0=vr0dc8meaabaqaciaacaGaaeqabaWaaeGaeaaakeaaimaacqWFce=qaaa@3825@_*F *_be *F *functional classes, not necessarily disjoint. One could use software like [25] or SAGE library tools (see, e.g., [26]) and public databases (e.g., Gene Ontology, Entrez Gene, Unigene cluster) to annotate and organize the expression values from a microarray experiment into families related by the biological characteristics of the genes or of their encoded proteins. Note that not all the genes can be functionally annotated and hence the set of all annotated genes C
 MathType@MTEF@5@5@+=feaafiart1ev1aaatCvAUfKttLearuWrP9MDH5MBPbIqV92AaeXatLxBI9gBamrtHrhAL1wy0L2yHvtyaeHbnfgDOvwBHrxAJfwnaebbnrfifHhDYfgasaacH8akY=wiFfYdH8Gipec8Eeeu0xXdbba9frFj0=OqFfea0dXdd9vqai=hGuQ8kuc9pgc9s8qqaq=dirpe0xb9q8qiLsFr0=vr0=vr0dc8meaabaqaciaacaGaaeqabaWaaeGaeaaakeaaimaacqWFce=qaaa@3825@ := ⋃ C
 MathType@MTEF@5@5@+=feaafiart1ev1aaatCvAUfKttLearuWrP9MDH5MBPbIqV92AaeXatLxBI9gBamrtHrhAL1wy0L2yHvtyaeHbnfgDOvwBHrxAJfwnaebbnrfifHhDYfgasaacH8akY=wiFfYdH8Gipec8Eeeu0xXdbba9frFj0=OqFfea0dXdd9vqai=hGuQ8kuc9pgc9s8qqaq=dirpe0xb9q8qiLsFr0=vr0=vr0dc8meaabaqaciaacaGaaeqabaWaaeGaeaaakeaaimaacqWFce=qaaa@3825@_*i *_⊂ G
 MathType@MTEF@5@5@+=feaafiart1ev1aaatCvAUfKttLearuWrP9MDH5MBPbIqV92AaeXatLxBI9gBamrtHrhAL1wy0L2yHvtyaeHbnfgDOvwBHrxAJfwnaebbnrfifHhDYfgasaacH8akY=wiFfYdH8Gipec8Eeeu0xXdbba9frFj0=OqFfea0dXdd9vqai=hGuQ8kuc9pgc9s8qqaq=dirpe0xb9q8qiLsFr0=vr0=vr0dc8meaabaqaciaacaGaaeqabaWaaeGaeaaakeaaimaacqWFge=raaa@382D@.

### Biological homogeneity index

Consider two annotated genes *x, y *that belong to the same statistical cluster D
 MathType@MTEF@5@5@+=feaafiart1ev1aaatCvAUfKttLearuWrP9MDH5MBPbIqV92AaeXatLxBI9gBamrtHrhAL1wy0L2yHvtyaeHbnfgDOvwBHrxAJfwnaebbnrfifHhDYfgasaacH8akY=wiFfYdH8Gipec8Eeeu0xXdbba9frFj0=OqFfea0dXdd9vqai=hGuQ8kuc9pgc9s8qqaq=dirpe0xb9q8qiLsFr0=vr0=vr0dc8meaabaqaciaacaGaaeqabaWaaeGaeaaakeaaimaacqWFdepraaa@3827@. Let us say that C
 MathType@MTEF@5@5@+=feaafiart1ev1aaatCvAUfKttLearuWrP9MDH5MBPbIqV92AaeXatLxBI9gBamrtHrhAL1wy0L2yHvtyaeHbnfgDOvwBHrxAJfwnaebbnrfifHhDYfgasaacH8akY=wiFfYdH8Gipec8Eeeu0xXdbba9frFj0=OqFfea0dXdd9vqai=hGuQ8kuc9pgc9s8qqaq=dirpe0xb9q8qiLsFr0=vr0=vr0dc8meaabaqaciaacaGaaeqabaWaaeGaeaaakeaaimaacqWFce=qaaa@3825@(*x*) is a functional class containing gene *x*. Similarly C
 MathType@MTEF@5@5@+=feaafiart1ev1aaatCvAUfKttLearuWrP9MDH5MBPbIqV92AaeXatLxBI9gBamrtHrhAL1wy0L2yHvtyaeHbnfgDOvwBHrxAJfwnaebbnrfifHhDYfgasaacH8akY=wiFfYdH8Gipec8Eeeu0xXdbba9frFj0=OqFfea0dXdd9vqai=hGuQ8kuc9pgc9s8qqaq=dirpe0xb9q8qiLsFr0=vr0=vr0dc8meaabaqaciaacaGaaeqabaWaaeGaeaaakeaaimaacqWFce=qaaa@3825@(*y*) contains gene *y*. We will assign the indicator function *I*(C
 MathType@MTEF@5@5@+=feaafiart1ev1aaatCvAUfKttLearuWrP9MDH5MBPbIqV92AaeXatLxBI9gBamrtHrhAL1wy0L2yHvtyaeHbnfgDOvwBHrxAJfwnaebbnrfifHhDYfgasaacH8akY=wiFfYdH8Gipec8Eeeu0xXdbba9frFj0=OqFfea0dXdd9vqai=hGuQ8kuc9pgc9s8qqaq=dirpe0xb9q8qiLsFr0=vr0=vr0dc8meaabaqaciaacaGaaeqabaWaaeGaeaaakeaaimaacqWFce=qaaa@3825@(*x*) = C
 MathType@MTEF@5@5@+=feaafiart1ev1aaatCvAUfKttLearuWrP9MDH5MBPbIqV92AaeXatLxBI9gBamrtHrhAL1wy0L2yHvtyaeHbnfgDOvwBHrxAJfwnaebbnrfifHhDYfgasaacH8akY=wiFfYdH8Gipec8Eeeu0xXdbba9frFj0=OqFfea0dXdd9vqai=hGuQ8kuc9pgc9s8qqaq=dirpe0xb9q8qiLsFr0=vr0=vr0dc8meaabaqaciaacaGaaeqabaWaaeGaeaaakeaaimaacqWFce=qaaa@3825@(*y*)) the value 1 if C
 MathType@MTEF@5@5@+=feaafiart1ev1aaatCvAUfKttLearuWrP9MDH5MBPbIqV92AaeXatLxBI9gBamrtHrhAL1wy0L2yHvtyaeHbnfgDOvwBHrxAJfwnaebbnrfifHhDYfgasaacH8akY=wiFfYdH8Gipec8Eeeu0xXdbba9frFj0=OqFfea0dXdd9vqai=hGuQ8kuc9pgc9s8qqaq=dirpe0xb9q8qiLsFr0=vr0=vr0dc8meaabaqaciaacaGaaeqabaWaaeGaeaaakeaaimaacqWFce=qaaa@3825@(*x*) and C
 MathType@MTEF@5@5@+=feaafiart1ev1aaatCvAUfKttLearuWrP9MDH5MBPbIqV92AaeXatLxBI9gBamrtHrhAL1wy0L2yHvtyaeHbnfgDOvwBHrxAJfwnaebbnrfifHhDYfgasaacH8akY=wiFfYdH8Gipec8Eeeu0xXdbba9frFj0=OqFfea0dXdd9vqai=hGuQ8kuc9pgc9s8qqaq=dirpe0xb9q8qiLsFr0=vr0=vr0dc8meaabaqaciaacaGaaeqabaWaaeGaeaaakeaaimaacqWFce=qaaa@3825@(*y*) match (in case of membership to multiple functional classes, any one match will be sufficient). As genes *x *and *y *are in the same statistical cluster, we expect the two functional classes to match. Thus, the following mathematical measure evaluates the biological similarity of the statistical clusters:

BHI=1k∑j=1k1nj(nj−1)∑x≠y∈DjI(C(x)=C(y)),
 MathType@MTEF@5@5@+=feaafiart1ev1aaatCvAUfKttLearuWrP9MDH5MBPbIqV92AaeXatLxBI9gBamrtHrhAL1wy0L2yHvtyaeHbnfgDOvwBHrxAJfwnaebbnrfifHhDYfgasaacH8akY=wiFfYdH8Gipec8Eeeu0xXdbba9frFj0=OqFfea0dXdd9vqai=hGuQ8kuc9pgc9s8qqaq=dirpe0xb9q8qiLsFr0=vr0=vr0dc8meaabaqaciaacaGaaeqabaWaaeGaeaaakeaacqWGcbGqcqWGibascqWGjbqscqGH9aqpdaWcaaqaaiabigdaXaqaaiabdUgaRbaadaaeWbqaamaalaaabaGaeGymaedabaGaemOBa42aaSbaaSqaaiabdQgaQbqabaGccqGGOaakcqWGUbGBdaWgaaWcbaGaemOAaOgabeaakiabgkHiTiabigdaXiabcMcaPaaaaSqaaiabdQgaQjabg2da9iabigdaXaqaaiabdUgaRbqdcqGHris5aOWaaabuaeaacqWGjbqscqGGOaakimaacqWFce=qcqGGOaakcqWG4baEcqGGPaqkcqGH9aqpcqWFce=qcqGGOaakcqWG5bqEcqGGPaqkcqGGPaqkaSqaaiabdIha4jabgcMi5kabdMha5jabgIGiolab=nq8enaaBaaameaacqWGQbGAaeqaaaWcbeqdcqGHris5aOGaeiilaWcaaa@68CC@

where *k *is the number of statistical clusters and for cluster D
 MathType@MTEF@5@5@+=feaafiart1ev1aaatCvAUfKttLearuWrP9MDH5MBPbIqV92AaeXatLxBI9gBamrtHrhAL1wy0L2yHvtyaeHbnfgDOvwBHrxAJfwnaebbnrfifHhDYfgasaacH8akY=wiFfYdH8Gipec8Eeeu0xXdbba9frFj0=OqFfea0dXdd9vqai=hGuQ8kuc9pgc9s8qqaq=dirpe0xb9q8qiLsFr0=vr0=vr0dc8meaabaqaciaacaGaaeqabaWaaeGaeaaakeaaimaacqWFdepraaa@3827@_*j*_, *n*_*j *_= *n*(D
 MathType@MTEF@5@5@+=feaafiart1ev1aaatCvAUfKttLearuWrP9MDH5MBPbIqV92AaeXatLxBI9gBamrtHrhAL1wy0L2yHvtyaeHbnfgDOvwBHrxAJfwnaebbnrfifHhDYfgasaacH8akY=wiFfYdH8Gipec8Eeeu0xXdbba9frFj0=OqFfea0dXdd9vqai=hGuQ8kuc9pgc9s8qqaq=dirpe0xb9q8qiLsFr0=vr0=vr0dc8meaabaqaciaacaGaaeqabaWaaeGaeaaakeaaimaacqWFdepraaa@3827@_*j *_∩ C
 MathType@MTEF@5@5@+=feaafiart1ev1aaatCvAUfKttLearuWrP9MDH5MBPbIqV92AaeXatLxBI9gBamrtHrhAL1wy0L2yHvtyaeHbnfgDOvwBHrxAJfwnaebbnrfifHhDYfgasaacH8akY=wiFfYdH8Gipec8Eeeu0xXdbba9frFj0=OqFfea0dXdd9vqai=hGuQ8kuc9pgc9s8qqaq=dirpe0xb9q8qiLsFr0=vr0=vr0dc8meaabaqaciaacaGaaeqabaWaaeGaeaaakeaaimaacqWFce=qaaa@3825@) is the number of annotated genes in D
 MathType@MTEF@5@5@+=feaafiart1ev1aaatCvAUfKttLearuWrP9MDH5MBPbIqV92AaeXatLxBI9gBamrtHrhAL1wy0L2yHvtyaeHbnfgDOvwBHrxAJfwnaebbnrfifHhDYfgasaacH8akY=wiFfYdH8Gipec8Eeeu0xXdbba9frFj0=OqFfea0dXdd9vqai=hGuQ8kuc9pgc9s8qqaq=dirpe0xb9q8qiLsFr0=vr0=vr0dc8meaabaqaciaacaGaaeqabaWaaeGaeaaakeaaimaacqWFdepraaa@3827@_*j*_, and where for a set *A*, *n*(*A*) denotes its size or cardinality.

This is a simple measure that is easy to interpret and implement once the reference collection of functional classes are in place. This also works with overlapping functional classes. This measure can be thought of as an average proportion of gene pairs with matched functional classes that are statistically clustered together based on their expression profiles.

### Biological stability index

Next we capture the stability of a clustering algorithm by inspecting the consistency of the biological results produced when the expression profile is reduced by one observational unit. This stability measure is unrelated to the one introduced by [3] which compared the clusters without regard to biological relevance.

In a microarray or SAGE study, each gene has an expression profile that can be thought of as a multivariate data value in ℜ^*p*^, for some *p *> 1. For example, in a time course microarray study, *p *could be the number of time points at which expression readouts were taken. In a two sample comparison, *p *could be the total (pooled) sample size, and so on. For each *i *= 1, 2,..., *p*, repeat the clustering algorithm for each of the *p *data sets in ℜ^*p*-1 ^obtained by deleting the observations at the *i*th position of the expression profile vectors. For each gene *g*, let D
 MathType@MTEF@5@5@+=feaafiart1ev1aaatCvAUfKttLearuWrP9MDH5MBPbIqV92AaeXatLxBI9gBamrtHrhAL1wy0L2yHvtyaeHbnfgDOvwBHrxAJfwnaebbnrfifHhDYfgasaacH8akY=wiFfYdH8Gipec8Eeeu0xXdbba9frFj0=OqFfea0dXdd9vqai=hGuQ8kuc9pgc9s8qqaq=dirpe0xb9q8qiLsFr0=vr0=vr0dc8meaabaqaciaacaGaaeqabaWaaeGaeaaakeaaimaacqWFdepraaa@3827@^*g,i*^, denote the cluster containing gene *g *in the clustering based on the reduced expression profile. Let D
 MathType@MTEF@5@5@+=feaafiart1ev1aaatCvAUfKttLearuWrP9MDH5MBPbIqV92AaeXatLxBI9gBamrtHrhAL1wy0L2yHvtyaeHbnfgDOvwBHrxAJfwnaebbnrfifHhDYfgasaacH8akY=wiFfYdH8Gipec8Eeeu0xXdbba9frFj0=OqFfea0dXdd9vqai=hGuQ8kuc9pgc9s8qqaq=dirpe0xb9q8qiLsFr0=vr0=vr0dc8meaabaqaciaacaGaaeqabaWaaeGaeaaakeaaimaacqWFdepraaa@3827@^*g*,0 ^be the cluster containing gene *g *using the full expression profile. For each pair of genes *x *and *y *in a biological class, we compare the statistical clusters containing *x *based on the original and the statistical cluster containing *y *based on the reduced profile. A stable clustering algorithm would produce similar answers, as judged biologically, based on the original and the reduced data. Thus, the clusters using full and reduced data, respectively, containing two functionally similar genes should have substantial overlaps. This is captured by the following stability measure and larger values of this index indicate more consistent answers:

BSI=1F∑i=1F1n(Ci)(n(Ci)−1)p∑j=1p∑x≠y∈Cin(Dx,0∩Dy,j)n(Dx,0)     (2)
 MathType@MTEF@5@5@+=feaafiart1ev1aaatCvAUfKttLearuWrP9MDH5MBPbIqV92AaeXatLxBI9gBamrtHrhAL1wy0L2yHvtyaeHbnfgDOvwBHrxAJfwnaebbnrfifHhDYfgasaacH8akY=wiFfYdH8Gipec8Eeeu0xXdbba9frFj0=OqFfea0dXdd9vqai=hGuQ8kuc9pgc9s8qqaq=dirpe0xb9q8qiLsFr0=vr0=vr0dc8meaabaqaciaacaGaaeqabaWaaeGaeaaakeaacqWGcbGqcqWGtbWucqWGjbqscqGH9aqpdaWcaaqaaiabigdaXaqaaiabdAeagbaadaaeWbqaamaalaaabaGaeGymaedabaGaemOBa4MaeiikaGccdaGae8NaXp0aaSbaaSqaaiabdMgaPbqabaGccqGGPaqkcqGGOaakcqWGUbGBcqGGOaakcqWFce=qdaWgaaWcbaGaemyAaKgabeaakiabcMcaPiabgkHiTiabigdaXiabcMcaPiabdchaWbaaaSqaaiabdMgaPjabg2da9iabigdaXaqaaiabdAeagbqdcqGHris5aOWaaabCaeaadaaeqbqaamaalaaabaGaemOBa4MaeiikaGIae83aXt0aaWbaaSqabeaacqWG4baEcqGGSaalcqaIWaamaaGcdaafaaqaaiab=nq8enaaCaaaleqabaGaemyEaKNaeiilaWIaemOAaOgaaOGaeiykaKcaleqabeqdcqWIPissaaGcbaGaemOBa4MaeiikaGIae83aXt0aaWbaaSqabeaacqWG4baEcqGGSaalcqaIWaamaaGccqGGPaqkaaaaleaacqWG4baEcqGHGjsUcqWG5bqEcqGHiiIZcqWFce=qdaWgaaadbaGaemyAaKgabeaaaSqab0GaeyyeIuoaaSqaaiabdQgaQjabg2da9iabigdaXaqaaiabdchaWbqdcqGHris5aOGaaCzcaiaaxMaadaqadaqaaiabikdaYaGaayjkaiaawMcaaaaa@8486@

A successful clustering is characterized by high values of both of these indices. The following subsection describes how to attribute a *p*-value to an observed index *I *for a given clustering algorithm by comparing it with random clustering of genes into the same number of clusters.

### Statistical scoring

By comparing with "random clustering", we can compute the observed level of significance or *p*-value for he above measures and a given clustering algorithm. This can be done by the following Monte Carlo steps:

Step 1. Compute a performance measure *I *for the clustering procedure under consideration.

Step 2. Compute the same performance measure *I *= *I*_*obs *_corresponding to a random clustering algorithm that ignores the data and assigns genes to clusters randomly and independently. This can easily be done by generating (*p *+ 1) independent simple random samples (with replacement) of size *M *out of {1,..., *k*}, where *k *denotes the desired number of clusters, and making the cluster assignments D
 MathType@MTEF@5@5@+=feaafiart1ev1aaatCvAUfKttLearuWrP9MDH5MBPbIqV92AaeXatLxBI9gBamrtHrhAL1wy0L2yHvtyaeHbnfgDOvwBHrxAJfwnaebbnrfifHhDYfgasaacH8akY=wiFfYdH8Gipec8Eeeu0xXdbba9frFj0=OqFfea0dXdd9vqai=hGuQ8kuc9pgc9s8qqaq=dirpe0xb9q8qiLsFr0=vr0=vr0dc8meaabaqaciaacaGaaeqabaWaaeGaeaaakeaaimaacqWFdepraaa@3827@^*g*,0 ^and D
 MathType@MTEF@5@5@+=feaafiart1ev1aaatCvAUfKttLearuWrP9MDH5MBPbIqV92AaeXatLxBI9gBamrtHrhAL1wy0L2yHvtyaeHbnfgDOvwBHrxAJfwnaebbnrfifHhDYfgasaacH8akY=wiFfYdH8Gipec8Eeeu0xXdbba9frFj0=OqFfea0dXdd9vqai=hGuQ8kuc9pgc9s8qqaq=dirpe0xb9q8qiLsFr0=vr0=vr0dc8meaabaqaciaacaGaaeqabaWaaeGaeaaakeaaimaacqWFdepraaa@3827@^*g,i*^, 1 ≤ *g *≤ *M*, 1 ≤ *i *≤ *p *accordingly. Denote the resulting value of the performance measure I*.

Step 3. Repeat Step 2 a large number of times, say *B*, yielding I1∗,⋯,IB∗
 MathType@MTEF@5@5@+=feaafiart1ev1aaatCvAUfKttLearuWrP9MDH5MBPbIqV92AaeXatLxBI9gBaebbnrfifHhDYfgasaacH8akY=wiFfYdH8Gipec8Eeeu0xXdbba9frFj0=OqFfea0dXdd9vqai=hGuQ8kuc9pgc9s8qqaq=dirpe0xb9q8qiLsFr0=vr0=vr0dc8meaabaqaciaacaGaaeqabaqabeGadaaakeaacqWGjbqsdaqhaaWcbaGaeGymaedabaGaey4fIOcaaOGaeiilaWIaeS47IWKaeiilaWIaemysaK0aa0baaSqaaiabdkeacbqaaiabgEHiQaaaaaa@36CF@.

Step 4. Compute the p-value as the proportion of times the performance measure by random cluster assignments exceeds (or equals to) the value obtained using the clustering algorithm under consideration

p=B−1∑s=1BI(Is∗≥Iobs).
 MathType@MTEF@5@5@+=feaafiart1ev1aaatCvAUfKttLearuWrP9MDH5MBPbIqV92AaeXatLxBI9gBaebbnrfifHhDYfgasaacH8akY=wiFfYdH8Gipec8Eeeu0xXdbba9frFj0=OqFfea0dXdd9vqai=hGuQ8kuc9pgc9s8qqaq=dirpe0xb9q8qiLsFr0=vr0=vr0dc8meaabaqaciaacaGaaeqabaqabeGadaaakeaacqWGWbaCcqGH9aqpcqWGcbGqdaahaaWcbeqaaiabgkHiTiabigdaXaaakmaaqahabaGaemysaKKaeiikaGIaemysaK0aa0baaSqaaiabdohaZbqaaiabgEHiQaaakiabgwMiZkabdMeajnaaBaaaleaacqWGVbWBcqWGIbGycqWGZbWCaeqaaOGaeiykaKcaleaacqWGZbWCcqGH9aqpcqaIXaqmaeaacqWGcbGqa0GaeyyeIuoakiabc6caUaaa@4795@

This proportion estimates the probability of obtaining a value as high as *I*_*obs *_just by chance (i.e., by "random clustering"). A 95% upper limit of the distribution of *I *under random clustering can be estimated by I([0.95B])∗
 MathType@MTEF@5@5@+=feaafiart1ev1aaatCvAUfKttLearuWrP9MDH5MBPbIqV92AaeXatLxBI9gBaebbnrfifHhDYfgasaacH8akY=wiFfYdH8Gipec8Eeeu0xXdbba9frFj0=OqFfea0dXdd9vqai=hGuQ8kuc9pgc9s8qqaq=dirpe0xb9q8qiLsFr0=vr0=vr0dc8meaabaqaciaacaGaaeqabaqabeGadaaakeaacqWGjbqsdaqhaaWcbaGaeiikaGIaei4waSLaeGimaaJaeiOla4IaeGyoaKJaeGynauJaemOqaiKaeiyxa0LaeiykaKcabaGaey4fIOcaaaaa@37EC@ where I(j)∗
 MathType@MTEF@5@5@+=feaafiart1ev1aaatCvAUfKttLearuWrP9MDH5MBPbIqV92AaeXatLxBI9gBaebbnrfifHhDYfgasaacH8akY=wiFfYdH8Gipec8Eeeu0xXdbba9frFj0=OqFfea0dXdd9vqai=hGuQ8kuc9pgc9s8qqaq=dirpe0xb9q8qiLsFr0=vr0=vr0dc8meaabaqaciaacaGaaeqabaqabeGadaaakeaacqWGjbqsdaqhaaWcbaGaeiikaGIaemOAaOMaeiykaKcabaGaey4fIOcaaaaa@31F2@ is the *j*th ordered I*.

### Range of *k*, the number of clusters

In general, the users will have the flexibility of investigating the performance of a clustering algorithm over a range of cluster numbers of their choosing. Some clustering algorithms such as Fanny or Model based clustering use data based selection of total number of hard clusters even if a larger number of clusters are desired by the user. For others, this choice is subjective. Often times, the biologists conducting the microarray experiment will make this call. For our illustration with the yeast data we have selected a range of *k *values around *k *= 7 which was used in the original analysis by [27].

### Human breast cancer progression data

We illustrate our methods using the expression profiles of 258 genes (SAGE tags) that were judged to be significantly differentially expressed at 5% significance level between four normal and seven ductal carcinoma in situ (DCIS) samples [26]. [26] combined various normal and tumor SAGE libraries in the public domain with their own SAGE libraries and used a modified form of *t*-statistics to compute *p-*values. Further details can be obtained from their paper and its supplementary web-site.

For constructing the functional classes, we have used a publicly available web-tool called AmiGO [25]. We were able to annotate 113 SAGE tags into the following eleven functional classes based on their primary biological functions. They were as follows: cell organization and biogenesis (24), transport (7), cell communication (15), cellular metabolism (48), cell cycle (6), cell motility (7), immune response (7), cell death(7), development (5), cell differentiation (5), cell proliferation (5), where the numbers in parentheses were the numbers of SAGE tags in a class. There were 23 genes that belonged to more than one functional class.

### Yeast sporulation data

As a second illustrative data set, we use a well known data set collected by [27]. This data set records expression profiles during the sporulation of budding yeast at seven time points. The original data set was filtered using the same criterion as in [27]. For our illustration, we look at a further subset of 513 genes (ORF's to be correct) that satisfy ∑ log expression ratio > 0, where the sum is over all the time points. Note that a positive value of the log of the expression ratio at a time point implies that the gene is positively expressed at that time and thus, in a sense, this is a collection of genes whose expression values change in a positive direction overall during the course of the experiment.

We use two separate web-based tools both using the GO ontology to annotate these ORF's. The resulting functional classifications were different although they had some common GO terms. We wanted to see whether the end comparison of the clustering algorithms is sensitive to the choice of the biological classes. To this end, we wanted to compare two different sets of functional classes, both based on the biological processes, with the same set of yeast ORF's.

For the first set of functional classes we mined the yeast genome database using the FatiGO webtool [28] at [29]. We have used the default FatiGO "level 3" GO terms. However it resulted in some very broad functional classes such as "cellular process" or "cellular physiological processes". In the end, we took a subset of the resulting terms which we judged to be more specific. This resulted in 295 annotated genes into the following ten overlapping biological classes: reproduction (14), cell communications (8), sex determination (4), metabolism (197), morphogenesis (13), cell differentiation (48), cell growth (7), cell regulation (85), response to stimulus (37) and localization (51).

The next set of functional classes were obtained using the web-based GO mining tool FunCat [30] available at [31] which did not offer a choice of "level" of the GO terms. Overall, 503 of the 513 genes were annotated into the following seventeen functional classes: metabolism (138), energy (27), cell cycle and DNA processing (152), transcription (50), protein synthesis (10), protein fate (72), protein with binding function or cofactor requirement (81), protein activity regulation (16), transport (63), cell communication (12), defense (36), interaction with environment (33), cell fate (17), development (41), biogenesis (77), cell differentiation (82).

### The clustering algorithms

We consider the following well known clustering algorithms representing the vast spectrum of clustering techniques that are available in statistical pattern recognition and machine learning literature. We evaluate these algorithms using the two biological performance measures BHI and BSI. One minus correlation was taken as the dissimilarity measure for the "distance" based algorithms. In addition, for UPGMA, Diana, Fanny, we also considered the standard Euclidean distance between expression vectors as a dissimilarity measure. Thus, overall, ten clustering schemes were subjected to this comparative evaluation.

#### UPGMA

This is perhaps the most commonly used clustering method with microarray data sets. This is an agglomerative hierarchical clustering algorithm [4] yielding a dendrogram that can be cut at a chosen height to produce the desired number of clusters. It uses a dissimilarity matrix in order to decide if two expression profiles are close or not.

#### K-means

K-means [6] is a partitioning method that is not hierarchical in nature. This algorithm uses a minimum "within-class sum of squares from the centers" criterion to select the clusters. The number of clusters needs to be fixed in advance.

#### Diana

This is also a hierarchical algorithm which is divisive in nature [7]. Thus at each level, a bigger cluster is divided into two smaller clusters that are furthest apart.

#### Fanny

This algorithm produces a fuzzy cluster [7]. Thus, a probability vector for each observation is reported that represents the probability of its cluster membership. A hard cluster can be produced by assigning it to the cluster with highest probability.

#### SOM

Clustering by self-organizing maps [8] is a popular method amongst the computational biologists and machine learning researchers. SOM is based on neural networks and can be regarded as a data visualization technique.

#### Model based clustering

Under this scheme [10], a statistical model is fit to the data. The model is a finite mixture of Gaussian distributions. Each mixture component represents a cluster. The Maximum likelihood method (EM algorithm) is used to fit the group membership and the mixture components. A number of Gaussian component models are compared as well. The number of clusters and the Gaussian models are chosen by the minimum BIC criterion.

#### SOTA

Self-organising tree algorithm or SOTA has received a great deal of attention in recent years and was used to cluster microarray gene expression data in [32]. Originally proposed by [9] for phylogenetic reconstruction, SOTA produces a divisive hierarchical binary tree structure using a neural network. It uses a fast algorithm and hence is suitable for clustering a large number of objects.

UPGMA (hclust) and K-Means are available in the base distribution of R. Diana and Fanny are available in the library "cluster". Model based clustering is available in the R-package mclust. For SOM, we have used an R code written by Niels Waller and Janine Illian [33]. For SOTA, we have used the MeV component of the TM4 package [34]. Servers running SOTA are also available ([35,36]).

## Authors' contributions

Susmita Datta: Development of statistical methods, identification of data sets and biological commentary; Somnath Datta: Development of statistical methods and computing. Both authors approved the final manuscript.

## Supplementary Material

Additional File 1 R-CODE FOR BHI AND BSI. The file contains an R-CODE for calculating the performance indices for clustering algorithms introduced in this paper. Click here for file
